# Expression of apoplast-targeted plant defensin *MtDef4.2* confers resistance to leaf rust pathogen *Puccinia triticina* but does not affect mycorrhizal symbiosis in transgenic wheat

**DOI:** 10.1007/s11248-016-9978-9

**Published:** 2016-08-31

**Authors:** Jagdeep Kaur, John Fellers, Alok Adholeya, Siva L. S. Velivelli, Kaoutar El-Mounadi, Natalya Nersesian, Thomas Clemente, Dilip Shah

**Affiliations:** 1 Donald Danforth Plant Science Center, St. Louis, MO 63132 USA; 2 USDA-ARS-HWWGRU, Department of Plant Pathology, Kansas State University, Manhattan, KS 66506 USA; 3 Mycorrhizal Applications, 1005 North Warson Road, BRDG Park, St. Louis, MO 63132 USA; 4 Center for Biotechnology, University of Nebraska-Lincoln, Lincoln, NE 68588 USA; 5 Department of Agronomy and Horticulture/Center for Plant Science Innovation, University of Nebraska-Lincoln, Lincoln, NE 68588 USA; 6 Department of Biology, Kutztown University of Pennsylvania, Kutztown, PA 19530 USA

**Keywords:** Plant defensin, MtDEF4.2, Leaf rust, *Puccinia triticina*, Genetic engineering, *Rhizophagus irregularis*

## Abstract

Rust fungi of the order *Pucciniales* are destructive pathogens of wheat worldwide. Leaf rust caused by the obligate, biotrophic basidiomycete fungus *Puccinia triticina* (*Pt*) is an economically important disease capable of causing up to 50 % yield losses. Historically, resistant wheat cultivars have been used to control leaf rust, but genetic resistance is ephemeral and breaks down with the emergence of new virulent *Pt* races. There is a need to develop alternative measures for control of leaf rust in wheat. Development of transgenic wheat expressing an antifungal defensin offers a promising approach to complement the endogenous resistance genes within the wheat germplasm for durable resistance to *Pt*. To that end, two different wheat genotypes, Bobwhite and Xin Chun 9 were transformed with a chimeric gene encoding an apoplast-targeted antifungal plant defensin MtDEF4.2 from *Medicago truncatula*. Transgenic lines from four independent events were further characterized. Homozygous transgenic wheat lines expressing MtDEF4.2 displayed resistance to *Pt* race MCPSS relative to the non-transgenic controls in growth chamber bioassays. Histopathological analysis suggested the presence of both pre- and posthaustorial resistance to leaf rust in these transgenic lines. MtDEF4.2 did not, however, affect the root colonization of a beneficial arbuscular mycorrhizal fungus *Rhizophagus irregularis*. This study demonstrates that the expression of apoplast-targeted plant defensin MtDEF4.2 can provide substantial resistance to an economically important leaf rust disease in transgenic wheat without negatively impacting its symbiotic relationship with the beneficial mycorrhizal fungus.

**Electronic supplementary material:**

The online version of this article (doi:10.1007/s11248-016-9978-9) contains supplementary material, which is available to authorized users.

## Introduction

Wheat is a major food crop grown on about 225 million hectares globally and provides 20 % of the worldwide caloric consumption (http://faostat3.fao.org/). Approximately, 700 million tons of wheat is produced annually with almost half produced in developing countries. However, wheat production must be increased to provide the calories needed for an ever increasing human population. Fungal pathogens cause significant losses of yield in all wheat-producing countries. In particular, rust pathogens of the order *Pucciniales* pose a major threat to food security around the world (Hulbert and Pumphrey 2014). *Puccinia triticina (Pt)* Eriks, causal agent of wheat leaf rust, is a major threat to wheat production in North America (Kolmer and Hughes 2013). Yield losses of up to 50 % can occur during epidemics (Huerta-Espino et al. 2011) and to combat yield losses wheat breeders use both major and minor resistance genes. Unfortunately, new rust races evolve rapidly limiting the effectiveness of major genes and forcing breeders to find new sources of resistance (Singh et al. 2011). On the other hand, while minor gene resistance is more durable, resistance is more quantitative and difficult to identify and transfer into an adapted cultivar (Kolmer 2013). New sources of resistance are often found in the wild relatives of wheat, but transferring resistance to adapted cultivars can be difficult and time consuming. Ultimately, a more durable type of resistance is needed that can withstand new races of *Pt*.

*Pt* is a biotrophic obligate pathogen with a complex life cycle involving five distinct spore types on two distinct hosts (Kolmer 2013). On the wheat host, a urediniospore germinates, finds a stomate, forms an appresorium, and grows until it finds an internal mesophyll cell. The fungus penetrates the cell wall and invaginates but does not penetrate the plasma membrane. Haustoria are formed which secrete effector proteins to reprogram the cell for nutrient production and uptake, while suppressing host defenses (O’Connell and Panstruga 2006). For engineering durable quantitative resistance to leaf rust, a transgenic approach may be needed to either express proteins that interfere with the *Pt* life cycle or to inhibit fungal colonization or growth. Antifungal proteins have the potential to address this need when used in combination with minor resistance genes. Successful identification of such proteins offers the opportunity to develop widely adapted leaf rust resistant wheat cultivars (Ayliffe et al. 2008; Lowe et al. 2011; McIntosh and Pretorius 2011).

Small cysteine-rich antifungal plant defensins are potentially useful for engineering broad-spectrum resistance to fungal pathogens in transgenic crops (Kaur et al. 2011; de Coninck et al. 2013). Three-dimensional structures of plant defensins are highly conserved and consist of three antiparallel β-strands and one α-helix that are stabilized by four disulfide bonds. Although structurally similar, plant defensins are diverse in their amino acid sequences (Thomma et al. 2002; Lay and Anderson 2005). This sequence diversity likely contributes to the different biological functions of defensins (Carvalho Ade and Gomes 2009; Van der Weerden and Anderson 2013).

The majority of plant defensins are synthesized as precursor proteins containing a secretory signal peptide and a cationic mature defensin domain, categorized as class I defensins. Class I defensins are targeted to the apoplast, however, a few class II defensins that carry an additional carboxy-terminal proprotein are targeted to the vacuole (Lay et al. 2003, 2014). Certain cationic plant defensins exhibit growth inhibitory activity against various fungi and oomycetes at micromolar (μM) concentrations and this potent antifungal activity translates into disease resistance against these pathogens in transgenic plants (Kaur et al. 2011). In case of rust pathogens, it has been reported that *Nicotiana alata* defensins, NaD1 and NaD2, inhibit germination of urediniospores and mitigate germ tube growth and differentiation of *Puccinia* species, and confer resistance to crown rust in oat seedlings when applied as a foliar spray (Dracatos et al. 2013).

The *Medicago truncatula* genome contains 63 defensin genes with the potential to encode over 80 defensins through alternative splicing (Maróti et al. 2015). One member of this gene family, *MtDef4.2* (previously reported as *MtDef4, MTR_8g070770*), encodes a precursor protein consisting of a 29-amino acid signal peptide and a 47-amino acid mature defensin (Sagaram et al. 2011). There are six additional *MtDef4.2* homologs, namely *MtDef4.1* and *MtDef4.3*-*4.7* (Kaur et al. 2012). MtDEF4.2 carries a net charge of +6 and inhibits the growth of several filamentous fungi including *Fusarium graminearum* (*Fg*) *in vitro* (Ramamoorthy et al. 2007). MtDEF4.2 has been previously shown to be internalized by *Fg* hyphae and binds to phosphatidic acid (PA), a pleiotropic signaling phospholipid present in fungal cells (Sagaram et al. 2013). In transgenic *Arabidopsis thaliana*, expression of the apoplast-targeted MtDEF4.2 confers strong resistance to a biotrophic obligate oomycete *Hyaloperonospora arabidopsidis* (*Hpa*) causing the downy mildew disease. If MtDEF4.2 is localized to either the vacuole or endoplasmic reticulum, resistance is lost (Kaur et al. 2012). Based on this study, we have shown that MtDEF4.2 is effective in providing resistance to oomycete and fungal pathogens. Thus, the objective of current investigation was to determine whether MtDEF4.2 provides resistance to leaf rust in transgenic wheat, while maintaining the association with the beneficial arbuscular mycorrhizal fungus (AMF). Transgenic wheat lines expressing an apoplast-localized MtDEF4.2 were evaluated for resistance to leaf rust pathogen *Pt*. The results indicate that this protein not only reduces the overall symptoms of leaf rust infection, but also retards the growth and differentiation of the fungal structures in the infected wheat leaf. Moreover, expression of this protein has no adverse effect on the colonization of transgenic wheat by the beneficial symbiotic AMF, *Rhizophagus irregularis (Ri*).

## Materials and methods

### Wheat growth conditions

Transgenic and non-transgenic wheat lines grown at the Plant Growth Facility of the Donald Danforth Plant Science Center were planted in Pro-Mix BRK soil mix (Hummert International, Earth City, MO). Plants were grown in a growth chamber with a photoperiod of 16 h light/8 h dark, 23/16 °C day/night temperature, 50 % relative humidity (RH) and light intensity of 500 μmol m^−2^ s^−1^. For growth, development and seed production comparison T_6_ generation of transgenic and non-transgenic wheat lines were grown in the greenhouse at 20–22 °C during the day and 19–21 °C at night, 40–75 % RH and supplemental light when light intensity went below 300 μmol m^−2^ s^−1^ during 600–2200 h. Transgenic and non-transgenic wheat plants for *Pt* inoculation were grown at the USDA-ARS Hard Winter Wheat Genetics Research Unit, Department of Plant Pathology, Kansas State University, KS (Bruce et al. 2014).

### Construction of the MtDef4.2 expression vector

To obtain a high level expression of MtDEF4.2 in transgenic wheat, a 231 bp full-length *MtDef4.2* cDNA sequence was synthesized (GenScript Inc., NJ) using the monocot-preferred codons and cloned as a *Nco*I-*Xba*I fragment between the Tobacco etch virus (TEV) mRNA leader sequence and Cauliflower mosaic virus (CaMV) 35S polyadenylation signal in the pRTL2 vector. The 5′-*TEV mRNA leader/MtDef4.2/CaMV 35S polyA*-3’ cassette was removed from the pRTL2 vector as an *Xho*I-*Pst*I fragment. This restriction fragment and the 1.9 kb *Hind*III-*Sal*I fragment containing the maize *Ubi1A promoter/intron* were cloned into the unique *Hind*III and *Pst*I sites of the vector *pPZP212* (Koncz and Schell 1986). *Agrobacterium tumefaciens* strain C58C1/pMP90 was transformed with the pPZP212 vector containing the chimeric *MtDef4.2* gene for wheat transformation (Hajdukiewicz et al. 1994; Koncz and Schell 1986).

### Wheat transformation and genetic analysis of transgenic wheat lines

Immature embryos of wheat cultivars Bobwhite (BW) and Xin Chun 9 (XC9) were used for transformation using a modification of the protocol previously described by Cheng et al. (1997). Six primary BW and five primary XC9 transgenic events (T_0_) were obtained and selfed to generate T_1_ lines. Mendelian segregation ratios of the T_1_ lines were determined by PCR and tested for goodness of fit of a 3:1 segregation using Chi square analysis. Only transgenic lines with single insert locus were progeny tested using PCR in subsequent T_2_ generation to obtain homozygous lines. Transgenic lines were advanced to the T_6_ generation.

### RNA isolation and quantitative RT-PCR analyses

Leaf and root tissue was harvested from the homozygous transgenic wheat lines along with non-transgenic BW and XC9 controls that were grown to the 3-leaf stage. Total RNA was isolated using the mirVana™ isolation kit (Life Technologies, Carlsbad, CA) according to the protocol, and quantified using a Nanodrop 2000 spectrophotometer (Thermo Scientific, Waltham, MA). Contaminating DNA was removed using a TURBO DNA-free™ kit (Ambion, Life Technologies, Carlsbad, CA) following the manufacturer’s instructions. Two μg of total RNA was used for reverse transcription using the iScript™ cDNA synthesis kit (Bio-Rad, Foster City, CA) using the supplier’s protocol. The resulting cDNA was used as template in a 10 μl reaction containing 1x Choice Taq polymerase buffer (Denville Scientific Inc., Denville, NJ), 25 mM MgCl_2_, 2.5 mM dNTP mix, 0.5 μM each of the forward (5′- ACTCGTGTCCACCATCTTCGTGTT-3′) and (5′ ATGGGCCCTTGAACTTGTGGGATT 3′) reverse primers, 1x SYBR mix (Molecular Probes, Foster City, CA), 0.5 U of Choice Taq polymerase. PCR amplification conditions used were 95 °C for 3 min; 95 °C for 10 s; 58 °C for 15 s, and 72 °C for 20 s, followed by a melting curve program. Dissociation curve was set at 95 °C for 10 s and 65 °C for 5 s followed by a slow ramp from 65 to 95 °C in a CFX384 Touch™ real-time PCR detection System (Bio-Rad, Foster City, CA). Expression was normalized to the ADP-ribosylation factor gene *Ta2291* as an endogenous control (Paolacci et al. 2009). All primers used showed amplification efficiency between 80-120 % and data were analyzed using ΔΔCt (delta-delta-Ct) method.

### Leaf rust inoculations, sampling and scoring

All transgenic lines (T_6_ generation), non-transgenic BW and XC9 wheat lines were inoculated with the *Pt* race MCPSS in a growth chamber assay. Ten plants from each line were inoculated at the 3-leaf stage by suspending 5 mg urediniospores into 1 ml of Soltrol 170 (Phillips 66, Bartlesville, OK) and spraying onto the plants using an atomizer at 40 psi. After inoculation, plants were transferred to a dew chamber with 100 % humidity overnight at 18 °C. Subsequently, plants were moved back into the growth chamber at 16 h/8 h day/night at 18 ºC. The severity of rust symptoms was scored visually at 15 dpi (days post inoculation) by rating the infection-type (IT) of each line on a scale of 0–4 as described by (McIntosh et al. 1995). On this scale, IT 0 (pronounced as “naught”) indicates no visible symptoms; IT; (pronounced as “fleck”) indicates resistant response along with hypersensitive “flecks” and no uredinia; IT 1 is characterized by minute uredinia surrounded by distinct necrotic areas; IT 2 indicates small uredinia surrounded by chlorosis; IT 3 indicates medium-sized uredinia frequently surrounded by chlorosis and IT 4 indicates large uredinia usually without chlorosis. In addition, a ‘+’ (plus) or ‘−’ (minus) sign is used to indicate bigger or smaller size uredinia, respectively, within the scale. Photographs of the representative leaf from each transgenic and control line were taken at 15 dpi. This experiment was repeated twice with similar results.

### Histopathological observations of leaf rust inoculated plants

Inoculated leaves were collected at 2-, 4- and 15 dpi collected in 15 ml screw cap tubes containing 12 ml of 1 M potassium hydroxide (KOH) and 2 μl of Tween-20. The leaves were incubated at 37 ºC for 12–16 h at room temperature (RT). Leaves were subsequently rinsed three times in the neutralization buffer, 50 mM Tris, pH 7.5 and stained overnight with 20 μg ml^−1^ wheat germ agglutinin-Oregon Green^®^ 488 conjugate (Molecular Probes) prepared in the neutralization buffer. Leaves were gently washed with water and mounted on slides for microscopy. For 2- and 4 dpi time points, stained leaves were imaged using a Zeiss LSM 510 confocal microscope (Zeiss, Jena, Germany) with a 40X, 1.2 NA water immersion, W Koor, C-Apochromat objective. Detection of fluorescence was done using 488 nm excitation (argon gas laser) and a BP 500–550 barrier filter. Maximum intensity projections were made using Imaris software (Bitplane AG, Zurich, Switzerland). A fluorescence microscope (Nikon SMZ1500, Melville, NY, USA) equipped with a QImaging Retiga 1300 camera (QImaging, Vancouver, BC, Canada) was used to visualize *Pt* infected wheat leaves at 15 dpi.

### Colonization of transgenic wheat lines by the AMF *Ri*

For testing the colonization of transgenic wheat lines by the arbuscular mycorrhizal fungus *Rhizophagus irregularis (Ri)*, seeds were surface sterilized with 20 % sodium hypochlorite (1.2 % active chlorine) containing a drop of Tween-20 (2 drops/1 ml) for 15 min in 50 ml falcon tubes. The seeds were then washed thoroughly with sterile water five times, and dried between folds of paper towel. Seeds were transferred to petri dishes containing wet filter paper and kept at RT for germination. After about a week, the germinated seedlings were transplanted into 101.6 mm pots containing 1:1 mixture of Profile and sand (obtained from Hummert International, Earth City, MO, USA) and inoculated with 200 propagules of a mixture of *Ri* prepared in natural clay calcite. Mock inoculations were done in a similar way with only calcite. Following inoculation, seedlings were grown in the greenhouse under the conditions described above. The plants were watered using reverse osmosis water when the moisture level went below 40 % as measured with humidity meter (Soil Master™, Mosser Lee Company, Millston, WI, USA). At 36 dpi, the plants were phenotyped for leaf number/plant and height. To assess the colonization, roots were harvested and stained with India ink as reported in Vierheilig et al. (1998). The harvested roots were cleared in 5 % potassium hydroxide by incubating at 70 °C in a water bath for 45 min. The roots were rinsed 3X with water and treated with 1 % hydrochloric acid at RT. The roots were washed with water and stained with 2 % India ink-vinegar solution overnight at RT. Next day the roots were washed with water 3X and destained in 5 % acetic acid for 1 h. Final destaining was done in 50 % lacto-glycerol overnight. The roots were cut in small segments and mounted on glass slides using 50 % lacto-glycerol. AMF colonization assessments were done by the magnified line intersect method (McGonigle et al. 1990) and 200 root segments/line were scored to measure percent colonization. Data were analyzed using student’s *t* test.

## Results

### Generation of transgenic wheat lines expressing apoplast-targeted MtDEF4.2

**Fig. 1:**
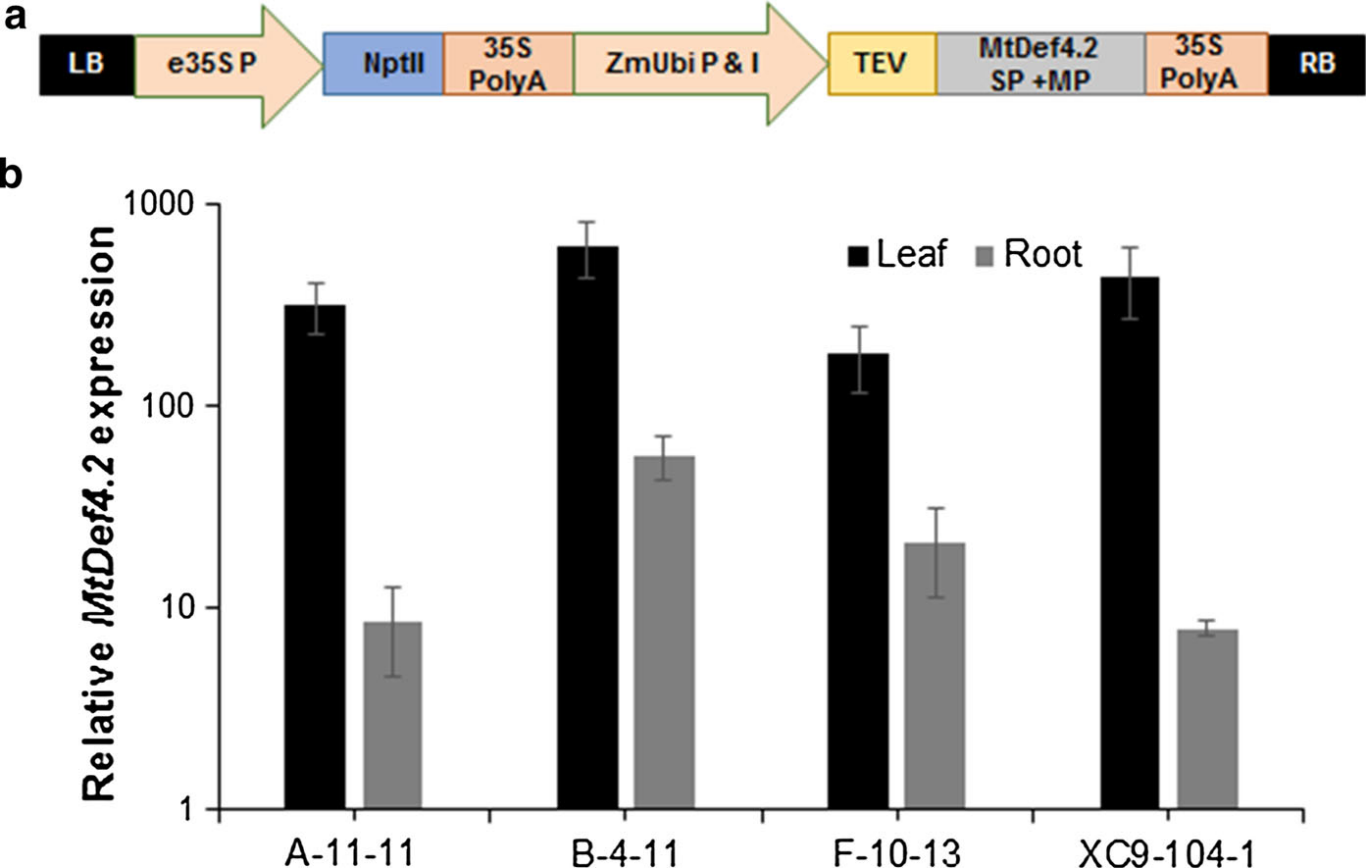
Chimeric *MtDef4.2* gene expression cassette and its expression in transgenic wheat. **a** The monocot codon-optimized *MtDef4*.2 was placed under the control of the constitutively expressing maize *Ubi1A* promoter/intron/tobacco etch virus (TEV) mRNA leader sequence and 3′ polyadenylation CaMV35S signal terminates transcription. Plant selectable marker gene *NPTII* is driven by enhanced CaMV35S promoter and 35S polyadenylation is transcription termination sequence. *LB* left border; *P* promoter; *NPTII* neomycin phosphotransferase II; *I* intron; *SP* signal peptide; *MP* mature peptide; *RB* right border. **b** Quantitative RT-PCR of *MtDef4.2* in the leaf and root tissue of various transgenic wheat lines. Relative expression levels of *MtDef4.2* are reported in logarithmic scale and were normalized to expression of wheat ADP glycosylation gene *Ta2291*. *Error bars* represent SE of three different biological replicates

In our previously published study, we observed that MtDEF4.2 targeted to the apoplast of transgenic *Arabidopsis* conferred resistance to the biotrophic obligate oomycete *Hpa* (Kaur et al. 2012). We therefore sought to determine if MtDEF4.2 was effective in providing resistance to an economically important basidiomycete biotrophic obligate fungal pathogen *Pt* in wheat. For constitutive expression of this protein in transgenic wheat, the full-length *MtDef4.2* gene was placed under the control of the maize *Ubi1A* promoter (Christensen et al. 1992; Fig. 1a) and introduced into two different spring varieties, BW and XC9.

Six independent transgenic BW T_0_ events and five XC9 T_0_ events were generated. Ten to 15 T_1_ generation families from each of the transgenic events 431-1-3-1 (BW-A), 431-5-1-1 (BW-B), 431-5-2-1 (BW-C), 445-2-1-1 (BW-D), 445-2-2-1 (BW-E), 446-1-1-1 (BW-F), 426-2-1-1 (XC9-101), 426-2-1-2 (XC9-102), 426-2-1-3 (XC9-103) and 426-2-1-5 (XC9-104) were tested for the presence of the *MtDef4.2* gene using PCR. Based on the χ^2^ analysis, transgenic wheat lines BW-A, BW-B, BW-F and XC9-104 inherited *MtDef4.2* as a single locus (Supplementary Table 1). Only single locus transgenic lines were advanced to the T_2_ generation for progeny testing using PCR, and homozygous lines BW-A-11, BW-B-4, BW-F-10, and XC9-104-1 were identified (Supplementary Table 2). Homozygous transgenic lines were advanced to the T_6_ generation through self-pollination.

### Expression analysis of the *MtDef4.2* in transgenic wheat lines

Transgenic lines BW-A-11, BW-B-4, BW-F-10 and XC9-104-1 showed 319-, 628-, 182- and 442-fold expression of *MtDef4.2* mRNA in leaves relative to the housekeeping gene *Ta2291* (Fig. 1b). Transgenic wheat lines BW-B-4 and XC9-104-1 had the highest expression of *MtDef4.2* mRNA in the leaf tissue. The expression of *MtDef4*.2 mRNA in roots, however, was significantly lower than in leaves (Fig. 1b). Transgenic lines BW-A-11, BW-B-4, BW-F-10 and XC9-104-1 were determined to have 9-, 57-, 28- and 8 fold expression. Thus, highest expression of *MtDef4*.2 mRNA was observed in the roots of transgenic lines BW-B-4 and BW-F-10.

### Leaf rust resistance phenotyping of transgenic wheat lines expressing *MtDef4.2*

**Fig. 2:**
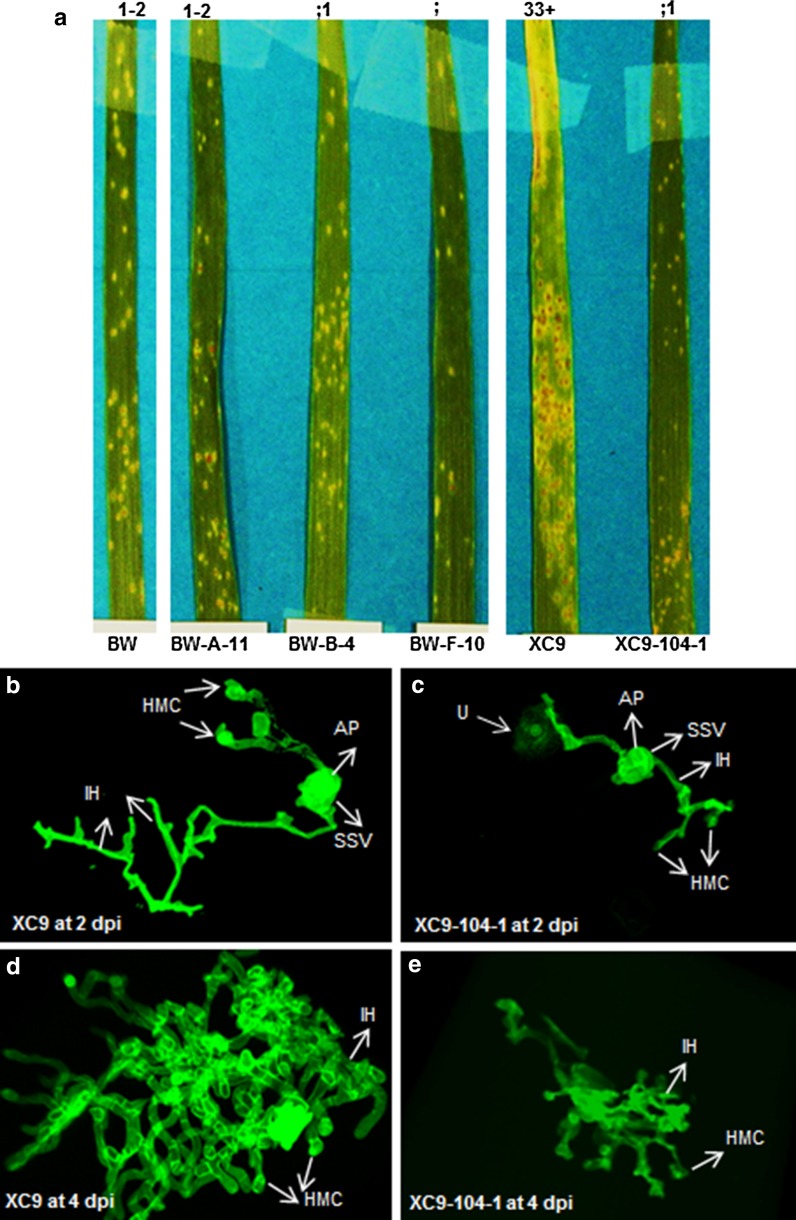
Photographs and confocal images of the representative leaves of non-transgenic and transgenic wheat lines infected with *Pt* race MCPSS. **a** Plants were scored for visual symptoms at 15 dpi and disease ratings were done on 0–4 scale as described in McIntosh et al. (1995). Average IT rating is listed on the *top* of each leaf. **b** Confocal microscopic image of XC9 at 2 dpi. **c** XC9-104-1 at 2 dpi. **d** XC9 at 4 dpi. **e** XC9-104-1 at 4 dpi. Between 10–15 leaf segments with infection foci were scanned. *U* urediniospore; *GT* germ tube; *AP* appresorium; *HMC* haustoria mother cell; *SSV* substomatal vesicle; *IH* infection hypha. *Scale bar* = 50 μm

Wheat lines were challenged at the 3-leaf stage with the *Pt* race MCPSS in the growth chamber. *Pt* race MCPSS was selected due to its ability to partially overcome the leaf rust resistance gene *Lr26* present in the wild type BW (Germán and Kolmer 2012). The inoculated seedlings were phenotyped for infection type (IT) on a 0–4 scoring scale at 15 dpi. Non-transgenic BW control challenged with *Pt* race MCPSS showed an IT score of 2 indicating a moderately resistant reaction. In comparison to BW, all three transgenic wheat lines BW-A-11, BW-B-4 and BW-F-10 were highly resistant with an IT score ranging from chlorosis to 1, especially BW-F-10 showed a hypersensitive fleck response (Table 1). Transgenic wheat line XC9-104-1 showed a highly resistant phenotype (IT ranging from fewer pustules to 1) compared to the non-transgenic control XC9 that was moderately susceptible (IT score of 3) (Table 1). The representative leaf from each transgenic and control line showing the symptoms of rust infection at 15 dpi is shown in Fig. 2a.

**Table 1:** Leaf rust phenotype on non-transgenic and transgenic wheat lines infected with *Pt* race MCPSS

Line name	Average infection type (IT) scores at 15 dpi^a^	Host reaction
BW	2	Moderately resistant
BW-A-11	Chlorosis, ;1	Highly resistant
BW-B-4	;1	Highly resistant
BW-F-10	Chlorosis, ;	Highly resistant
XC9	3	Moderately susceptible
XC9-104	Fewer pustules to ;1	Highly resistant

^a^Scored on 0–4 scale where *0* no visible uredinia, ; (*fleck*) hypersensitive response, *1* small uredinia with necrosis, *2* small to medium uredinia with green islands surrounded by necrosis or chlorosis, *3* medium uredinia with or without chlorosis, *4* large uredinia without chlorosis per McIntosh et al. (1995)

### Histopathological analysis of leaf rust resistance in transgenic wheat

**Fig. 3:**
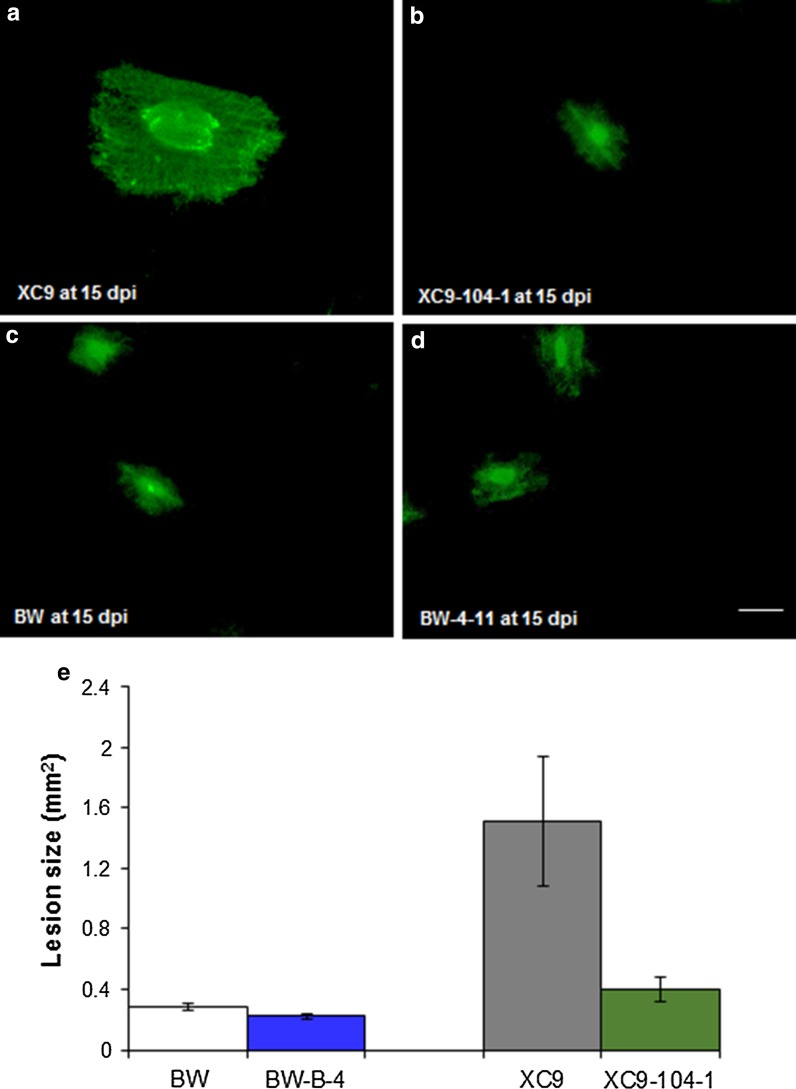
Representative fluorescence microscopic images of non-transgenic and transgenic wheat lines infected with *Pt* race MCPSS at 15 dpi. **a** XC9. **b** XC9-104-1. **c** BW. **d** BW-B-4. **e** Lesion size calculated using Image J. For lesion size measurements 12, 25, 19, 21 infection colonies for XC9, XC9-104-1, BW and B-4-11, respectively, were assessed. *Error bars* represent standard error of the means. Data were analyzed using Student’s *t* test, **** represents significance level at *P* < 0.0001. *Scale bar* 0.5 mm

Because *Pt* structural differentiation occurs within the first 6 dpi, we decided to look at 2- and 4 dpi time points to capture major differences between the highly resistant transgenic wheat line XC9-104-1 and its non-transgenic control line XC9 (Fig. 2a; Table 1) using confocal microscopy. At 2 dpi, both non-transgenic XC9 and transgenic line XC9-104-1 showed differentiation of various infection structures such as urediniospores (U), germ tube (GT), appresorium (AP), substomatal vesicle (SSV), haustorial mother cell (HMC) and infection hyphae (IH); however, XC9-104-1 line showed less proliferation of IH (Fig. 2b, c). At 4 dpi, while the XC9 control showed an elaborate network of HMC and IH (Fig. 2d), transgenic line XC9-104-1 showed dramatic reduction in the number of HMCs leading to IH (Fig. 2e) indicating slow progression of *Pt* infection. At 15 dpi, using fluorescence microscopy, we photographed the infection foci in these two lines and measured the lesion size. In agreement with the visual IT scores, lesions on the non-transgenic control XC9 leaf were dramatically bigger than those on the transgenic XC9-104-1 leaf (Fig. 3a, b; Table 1). XC9 had an average lesion area of 1.5 ± 0.40 mm^2^ whereas XC9-104-1 had an average lesion area of only 0.4 ± 0.07 mm^2^ (Fig. 3e). For comparison, we measured the lesion size of transgenic wheat event B-4-11 (area of 0.3 ± 0.02 mm^2^) and its corresponding non-transgenic control BW (0.2 ± 0.01 mm^2^), and found no significant difference between the two (Fig. 3c, d). Although in this experiment transgenic line B-4-11 showed reduced symptoms of *Pt* infection as indicated by an IT of ;1 vs an IT score of 1–2 for control BW (Table 1). Based on the temporal histopathological analyses of *Pt* infection in transgenic line XC9-104-1 expressing MtDEF4.2, it seems that both pre- and post-haustorial resistance contribute to the overall reduction of symptoms.

### Colonization of transgenic wheat by the beneficial arbuscular mycorrhizal fungus *Ri*

To determine if expression of MtDEF4.2 in transgenic wheat lines alters colonization by *Ri*, two transgenic wheat lines BW-B-4 and XC9-104-1 along with non-transgenic control lines BW and XC9 were tested. Leaf number/plant, plant height and percent colonization were measured at 36 dpi in two independent experiments, and average is reported in Table 2. Transgenic wheat line BW-B-4 showed plant height of 21 ± 2.41 cm and 15.25 ± 3.28 % colonization that were similar to non-transgenic control BW (20.70 ± 2.93 cm plant height and 14.45 ± 2.77 % colonization). In fact, BW-B-4 transgenic line had more leaves (6 ± 0.20 vs. 5 ± 0.20 leaves in the control BW) (Table 2). Similarly, transgenic line XC9-104-1 had 6 ± 0.60 leaves per plant, height of 15.4 ± 1.88 cm and 24.50 ± 6.03 % colonization which was similar to those of the non-transgenic control line XC9 with 7 ± 0.24 leaves/plant, 19.5 ± 3.75 cm height and 21.50 ± 4.83 % colonization (Table 2). Mock inoculated transgenic wheat line XC9-104-1 and its non-transgenic control XC9 had similar leaf number/ plant and plant height. However, transgenic line BW-B-4 had higher leaf number/plant (6 ± 0.17) and plant height (23.65 ± 0.72) than its non-transgenic line BW with leaf number/plant of 5 ± 0.00 and height of 21.37 ± 0.71 cm (Table 2). As expected, the mock-inoculated lines had no AMF colonization (Table 2). Importantly, this study demonstrates that the expression of an antifungal plant defensin MtDEF4.2 does not alter AMF symbiosis in transgenic wheat.

**Table 2:** Average AMF colonization of non-transgenic and transgenic wheat lines infected with *Ri* at 36 dpi from two independent experiments

Wheat line	Leaf number/ plant		Plant height (cm)		Percent colonization^a^	
	Mean ± SEM	*P* value	Mean ± SEM	*P* value	Mean ± SEM	*P* value
BW^b^	5 ± 0.20	–	21.00 ± 2.41	–	15.25 ± 3.28	–
BW-B-4^b^	6 ± 0.20	0.0341*	20.70 ± 2.93	0.9147	14.45 ± 2.77	0.8580
XC9^b^	7 ± 0.24	–	19.50 ± 3.75	–	21.50 ± 4.83	–
XC9-104-1^b^	6 ± 0.60	0.7990	15.4 ± 1.88	0.3998	24.50 ± 6.03	0.7024
BW Mock^c^	5 ± 0.00	–	21.37 ± 0.72	–	Not applicable	
BW-B-4 Mock^c^	6 ± 0.17	0.0005***	23.65 ± 0.72	0.0491*	Not applicable	
XC9 Mock^c^	6 ± 0.63	–	22.55 ± 1.12	–	Not applicable	
XC9-104-1 Mock^c^	6 ± 0.52	1.000	19.13 ± 1.39	0.1037	Not applicable	

^a^Mycorrhizal colonization includes the observations of internal hyphae, arbuscules and vesicles

^b^Eight plants were evaluated for treatment

^c^Six plants were evaluated for mock

*, *** Indicate significant difference at *P* = 0.05 and *P* = 0.001 in the student’s *t* test

### Effect of *MtDef4.2* expression on wheat development and seed traits

Transgenic wheat lines were grown alongside of non-transgenic controls for growth, development and yield parameters measurements. We did not observe any statistically significant difference in the seed germination rate and seedling emergence. At 2.5 weeks, vegetative growth of the transgenic wheat lines was not different from controls (Supplementary Fig. 1a). At 13 weeks, transgenic wheat lines BW-A-11, BW-B-4, BW-F-10, XC9-104-1 grew normally like controls as shown in Supplementary Fig. 1b. After harvesting, we measured the size of individual primary head, seed count/head and seed weight/head as described in Supplementary Table 3. At whole plant level, transgenic wheat lines looked similar to non-transgenic controls (Supplementary Fig. 2a). Primary head size of transgenic wheat lines BW-A-11 and BW-B-4 of 9.24 ± 0.02 cm and 9.14 ± 0.20 cm, respectively, was significantly bigger than the non-transgenic control BW of 8.56 ± 0.08 cm. However, the head size of BW-F-10 (8.40 ± 0.14 cm) was not different than that of BW (Supplementary Table 3). When compared with the non-transgenic control XC9 with head size of 8.98 ± 0.17 cm, transgenic line XC9-104-1 showed similar head size of 8.92 ± 0.15 cm (Supplementary Table 3, Supplementary Fig. 2b). There was no significant difference in the seed count/head of transgenic lines BW-A-11 (38.6 ± 4.50) and BW-F-10 (36.6 ± 3.39) except for BW-B-4 that had lower seed count/head of 27.6 ± 2.11 compared to control BW with 36.2 ± 2.56. In comparison to the XC9 control with 39.6 ± 2.66 seed count/head, transgenic line XC9-104-1 also had lower seed count/head of 28.0 ± 2.86 (Supplementary Table 3, Supplementary Fig. 2c). Transgenic wheat lines BW-A-11, BW-B-4, BW-F-10 has seed weight/head of 1.46 ± 0.18 g, 1.18 ± 0.07 g, 1.34 ± 0.13 g, respectively, that was not different than the control BW with the seed weight/head of 1.30 ± 0.08 g. However, transgenic wheat line XC9-104-1 had lower seed weight/head of 1.30 ± 0.12 g as compared to 1.80 ± 0.13 g in control line XC9 (Supplementary Table 3). Based on these data, we conclude that in the highest expressing transgenic lines, BW-B-4 and XC9-104-1 (Fig. 1b), expression of the *MtDef4.2* gene has negative effects on seed number and seed weight.

## Discussion

Plant defensin MtDEF4.2 targted to the apoplast, but not to the intracellular compartments, provides strong resistance to an oomycete obligate biotroph *Hpa* in transgenic *Arabidopsis* (Kaur et al. 2012). In the present study, we have translated these findings to an economically important crop and show that expression of MtDEF4.2 in transgenic wheat confers resistance to leaf rust disease caused by a basidiomycete obligate biotroph *Pt*. Pathogenicity assays performed in the growth chamber showed resistance to *Pt* race MCPSS in four independent transgenic wheat lines when compared to moderately resistant BW and moderately susceptible XC9 controls. All transgenic wheat lines showed highly resistant leaf rust response compared to the non-transgenic controls. In particular, transgenic wheat lines BW-F-10 and XC9-104-1 showed robust resistance to *Pt* compared to the non-transgenic controls, BW and XC9, respectively (Table 1). By translating the IT scores into actual disease percentage (Bariana et al. 2007), non-transgenic BW showed ~20–30 % disease whereas transgenic BW-F-10 showed only <5 % disease. Similarly, while moderately susceptible reaction of non-transgenic XC9 equates to 50–60 % disease, transgenic line XC9-104-1 displayed significant reduction in disease severity of <5 %. We did not observe a strict correlation between the level of *MtDef4*.2 expression and IT score in our transgenic lines. Thus, BW-F-10 line with low expression of the gene showed strong resistance to *Pt.* This lack of correlation between the antifungal gene expression and degree of resistance to a fungal pathogen has also been reported recently in transgenic rice line overexpressing *OsOSM1* gene encoding an osmotin protein (Xue et al. 2016). In this study, transgenic rice line expressing the *OsOSM1* gene at the highest level was not the most resistant line. The lack of correlation between fungal resistance and expression levels has also been noted in transgenic tobacco plants expressing the cysteine-rich insect defensin, heliomycin (Banzet et al. 2002). Although a constitutive maize *Ubi1A* promoter was used for expression of *MtDef4.2* in transgenic wheat, it is likely that its expression was not uniform in different cell types of the leaf tissue. Thus, low-expressor BW-F-10 may have high expression of *MtDef4.2* in the leaf cell types initially colonized by *Pt* providing high resistance to this pathogen.

*Pt* in the infected wheat leaf tissue undergoes an elaborate differentiation and infection structure formation. The U develops a GT that generates an AP over stomata to gain entry into wheat tissues. Subsequently, the fungus differentiates into a complex series of infection structures including SSV, primary IH and HMC. Haustoria develop from the HMC by breaching the host cell wall. The expanding haustorium is invaginated by the host plasma membrane and new membrane is synthesized. Haustorial cells are separated from the host cytoplasm only by the host cell membrane and extrahaustorial matrix (Szabo and Bushnell 2001; Mendgen and Hahn 2002). Thus, haustoria are not intracellular, but they serve as major feeding structures through which nutrients are taken up by the fungus and also serve as target sites for delivery of effector proteins into host cells for suppressing host defense (Garnica et al. 2014). Accordingly, resistance to leaf rust is either prehaustorial or posthaustorial (Niks and Dekens 1991). The germination of U leading into GT and the differentiation of AP into HMC comprise the ‘penetration phase’. While the differentiation of H and IH constitutes the ‘parasitic phase’, eventual pustule development on the leaf surface depicts the ‘sporulation phase’ (Voegele 2006). Transgenic line XC9-104-1 displayed stark differences in the penetration and parasitic growth phases of fungal differentiation at 2- and 4 dpi, compared to the XC9 control (Fig. 2b–e). These differences eventually led to much smaller pustules on XC9-104-1 (Fig. 3e), and a highly resistant IT. Based on these data, it seems that XC9-104-1 line conferred both pre- and posthaustorial resistance to leaf rust fungus *Pt*. A similar observation has been made in wheat cultivars carrying the leaf rust resistance genes where quantitative differences in structural differentiation of the fungus confer varying levels of resistance (Wang et al. 2013). In case of the hemibiotrophic fungal pathogen *Fg*, we have earlier shown that MtDEF4.2 rapidly permeabilizes fungal plasma membrane, gets internalized by the fungus and interacts with intracellular targets (Sagaram et al. 2013). It is likely that the apoplast-targeted MtDEF4.2 comes into direct contact with the *Pt* hyphae in the intercellular space and inhibits its further growth and differentiation by permeabilizing its plasma membrane and perhaps interacting with its intracellular targets. An immunofluorescence study employing antibodies generated against MtDEF4.2 might help determine if this protein is taken up by the cells of *Pt*. It has been reported that *N. alata* defensins, NaD1 and NaD2, inhibited *P. coronata* f. sp. *avenae* (*Pca*) and *P. sorghi* (*Ps*) urediniospore germination, germ tube growth and differentiation *in vitro* (Dracatos et al. 2013). These authors observed tip swelling and cytoplasmic granulation of the urediniospores concluding that these defensins were fungicidal to *Pca* and *Ps*. However, in their study, stably transformed transgenic plants were not generated and thus no histopathological evidence was provided.

In transgenic wheat lines resistant to leaf rust, apoplastic MtDEF4.2 is ideally located to establish direct contact with the invading pathogen, gain entry into it and suppress its growth. Based on our current understanding of the mode of action of MtDEF4.2, we propose a model, shown in Supplementary Fig. 3, for the antifungal action of this protein against *Pt* in transgenic wheat. We have reported earlier that MtDEF4.2 is internalized by the cells of *Fg* and binds to a bioactive phospholipid second messenger PA (Sagaram et al. 2013). We propose that MtDEF4.2 is internalized by the cells of *Pt* and binds to the plasma membrane resident PA. This interaction with PA leads to destabilization of the plasma membrane of *Pt* causing changes in ion fluxes and perturbation of calcium homeostasis (Muñoz et al. 2014). Once internalized, MtDEF4.2 binds to specific intracellular targets and causes programmed cell death. For future studies, it will be important to identify all interacting partners of this defensin to decipher the complexity of its antifungal activity. Collectively, antifungal action of the apoplast-localized MtDEF4.2 leads to a dramatic decrease in the symptoms of rust infection in the leaves of transgenic wheat.

Mycorrhizal fungi comprise a significant component of natural sustainable agroecosystems and many plants including agriculturally important species like wheat depend upon these beneficial fungi for nutrition and moisture. For commercial development of transgenic crops expressing defensins, it is important to evaluate the effects of these proteins not only on fungal pathogens, but also on the mycorrhizal fungi. In this study, we did not observe any differences between the transgenic wheat lines BW-B-4 and XC9-104-1 expressing highest levels of *MtDef4.2* mRNA in comparison to their non-transgenic controls, BW and XC9, as for their ability to colonize *Ri* (Table 2). In an earlier study, it was reported that transgenic *Solanum melongena* plants expressing a plant defensin DmAMP1 showed no adverse effects on either pre-symbiotic hyphal growth or mycorrhizal colonization (Turrini et al. 2004). Plant defensins are PR proteins classified as PR-12 (van Loon et al. 2006). Several PR proteins expressed in transgenic tobacco plants also did not affect colonization by AMF (Vierheilig et al. 1995). It is thus likely that the beneficial mycorrhizal fungi are insensitive to the antifungal activity of some PR proteins including MtDEF4.2 (de Coninck et al. 2013). Expression of *MtDef4*.2 mRNA in roots of transgenic lines seems low especially since we have used a strong constitutive maize ubiquitin promoter. However, the activity of the maize ubiquitin promoter has been reported to be strong in many tissues but not all, and furthermore its activity varies with the growth and development of the plant (Cornejo et al. 1993). Importantly, expression of *MtDef4.2* in the roots of transgenic lines B-4-11 and F-10-13 did not negatively impact their ability to be colonized by the beneficial AMF.

Under the greenhouse conditions used, we observed no differences between transgenic and non-transgenic wheat lines in their early vegetative growth and development except that flowering in non-transgenic wheat line XC9 and its transgenic wheat line XC9-104-1 was delayed by a week compared to BW, which is attributed to their different genotypes. At harvest, two transgenic wheat lines BW-B-4 and XC9-104-1 with the highest levels of *MtDef4.2* expression had lower seed count/head and seed weight/head compared to non-transgenic BW and XC9, respectively (Supplementary Table 3). For the remaining transgenic lines, we observed no negative effects on seed count/head and seed weight/head. It is likely that overexpression of *MtDef4.2* above a certain threshold has some penalty on yield-associated traits. However, leaf rust resistant transgenic lines expressing this gene below this threshold are not affected in their development or seed traits. Collectively, our results demonstrate that *MtDef4.2* is a very useful antifungal gene for engineering resistance to economically important obligate biotrophic fungal pathogens in transgenic crops. For commercial application of this technology, it might be desirable moving to a tissue-specific or pathogen-inducible promoter to limit the expression of this defensin where needed in a transgenic crop (Kaur et al. 2011).

## Electronic supplementary material

Below is the link to the electronic supplementary material.

**Supplementary Fig.** **1.** Representative pictures depicting vegetative growth comparison of transgenic wheat lines BW-A-11, BW-B-4, BW-F-10 and XC9-104-1 in comparison to non-transgenic control lines BW and XC9. **a** At 2.5 weeks after planning. **b** At 7 weeks after planting. Supplementary material 1 (TIFF 475 kb)

**Supplementary Fig.** **2.** Representative pictures of transgenic wheat lines BW-A-11, BW-B-4, BW-F-10, XC9-104-1 and non-transgenic control lines BW and XC9 at the time of harvest. **a** At 13 weeks after planting. **b** Individual heads at 13 weeks after planting. **c** seed count/head at 13 weeks after planting. Supplementary material 2 (TIFF 548 kb)

**Supplementary Fig.** **3.** Proposed model depicting the antifungal action of apoplast-targeted MtDEF4.2 on the cells of *Pt* race MCPSS. MtDEF4.2 is secreted to the apoplast where it comes in contact with fungal cells, permeabilizes its plasma membrane and causes disruption of the calcium homeostasis. It gets internalized and interacts with the plasma membrane resident phosphatidic acid (PA). The role of PA binding in the plasma membrane permeabilization by MtDEF4.2 and/ or its internalization into fungal cells remain to be elucidated. MtDEF4.2 interacts with as yet unidentified intracellular targets resulting in the fungal cell killing. Supplementary material 3 (TIFF 61 kb)

Supplementary material 4 (DOCX 28 kb)

## Abbreviations

DNA: Deoxyribonucleic acid
RNA: Ribonucleic acid
qRT-PCR: Quantitative reverse transcription polymerase chain reaction
T-DNA: Transfer deoxyribonucleic acid
