# Supplementary figures and images for: Expression of apoplast-targeted plant defensin MtDef4.2 confers resistance to leaf rust pathogen Puccinia triticina but does not affect mycorrhizal symbiosis in transgenic wheat
Source: Transgenic Res. 2016 Aug 31;26(1):37–49. doi: 10.1007/s11248-016-9978-9 (PMC5243879; doi:10.1007/s11248-016-9978-9)

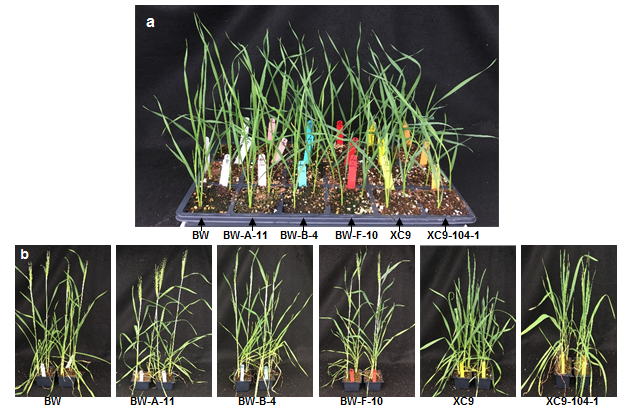

Supplement: Supplementary file 1 — Supplementary Fig. 1. Representative pictures depicting vegetative growth comparison of transgenic wheat lines BW-A-11, BW-B-4, BW-F-10 and XC9-104-1 in comparison to non-transgenic control lines BW and XC9. a At 2.5 weeks after planning. b At 7 weeks after planting. Supplementary material 1 (TIFF 475 kb) [file 11248_2016_9978_MOESM1_ESM.tif]

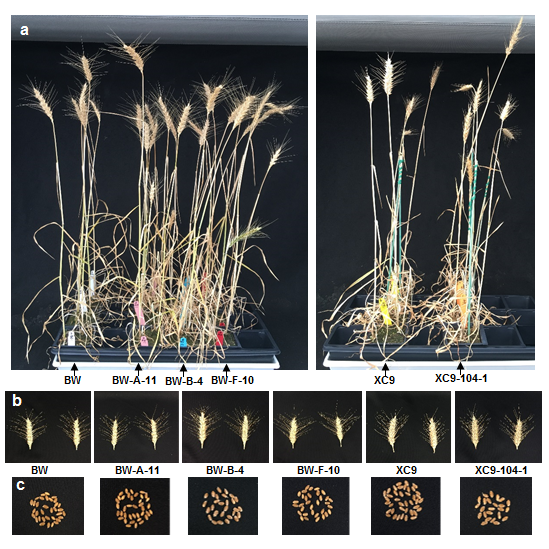

Supplement: Supplementary file 2 — Supplementary Fig. 2. Representative pictures of transgenic wheat lines BW-A-11, BW-B-4, BW-F-10, XC9-104-1 and non-transgenic control lines BW and XC9 at the time of harvest. a At 13 weeks after planting. b Individual heads at 13 weeks after planting. c seed count/head at 13 weeks after planting. Supplementary material 2 (TIFF 548 kb) [file 11248_2016_9978_MOESM2_ESM.tif]

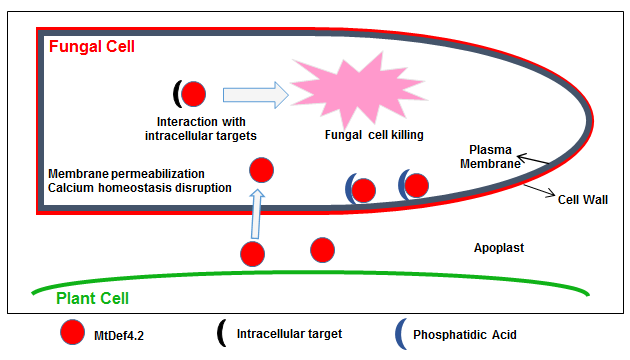

Supplement: Supplementary file 3 — Supplementary Fig. 3. Proposed model depicting the antifungal action of apoplast-targeted MtDEF4.2 on the cells of Pt race MCPSS. MtDEF4.2 is secreted to the apoplast where it comes in contact with fungal cells, permeabilizes its plasma membrane and causes disruption of the calcium homeostasis. It gets internalized and interacts with the plasma membrane resident phosphatidic acid (PA). The role of PA binding in the plasma membrane permeabilization by MtDEF4.2 and/ or its internalization into fungal cells remain to be elucidated. MtDEF4.2 interacts with as yet unidentified intracellular targets resulting in the fungal cell killing. Supplementary material 3 (TIFF 61 kb) [file 11248_2016_9978_MOESM3_ESM.tif]
