# Supplementary material for: Expression of apoplast-targeted plant defensin MtDef4.2 confers resistance to leaf rust pathogen Puccinia triticina but does not affect mycorrhizal symbiosis in transgenic wheat
Source: Transgenic Res. 2016 Aug 31;26(1):37–49. doi: 10.1007/s11248-016-9978-9 (PMC5243879; doi:10.1007/s11248-016-9978-9)
Supplement: Supplementary file 4 — Supplementary material 4 (DOCX 28 kb) [file 11248_2016_9978_MOESM4_ESM.docx]

**Supplementary Tables**

| **Supplementary Table 1.** Genetic characterization of T_1_ transgenic wheat events | | | | | | |
| --- | --- | --- | --- | --- | --- | --- |
| **Line #** | **Denoted by** | ***MtDef4*.2 PCR data** | | | **χ^2^ value** | **Insert number** |
|  |  | **Positive** | **Negative** | **Total # plants** | **(3:1)** |  |
| 431-1-3-1 | BW^1^-A | 14 | 1 | 15 | 2.68 | Single |
| 431-5-1-1 | BW-B | 9 | 6 | 15 | 1.80 | Single |
| 431-5-2-1 | BW-C | 15 | 0 | 15 | 5.00^*^ | Multiple |
| 445-2-1-1 | BW-D | 15 | 0 | 15 | 5.00^*^ | Multiple |
| 445-2-2-1 | BW-E | 14 | 0 | 14 | 4.66^*^ | Multiple |
| 446-1-1-1 | BW-F | 11 | 4 | 15 | 0.02 | Single |
| 426-2-1-1 | XC9^2^-101 | 15 | 0 | 15 | 5.00^*^ | Multiple |
| 426-2-1-2 | XC9-102 | 15 | 0 | 15 | 5.00^*^ | Multiple |
| 426-2-1-3 | XC9-103 | 15 | 0 | 15 | 5.00^*^ | Multiple |
| 426-2-1-5 | XC9-104 | 8 | 2 | 10 | 0.13 | Single |
| ^1^ Transgenic wheat generated in Bobwhite background  ^2^ Transgenic wheat generated in Xin Chun 9 background.^*^Significant at P = 0.05, chi-square ( **χ^2^**) table value at 1 d.f. = 3.84 | | | | | | |

| **Supplementary Table 2.** Identification of homozygous linages from single locus transgenic events in T_2_ generation | | | | | |
| --- | --- | --- | --- | --- | --- |
| **Line #** | **Plant #** | ***MtDef4.2* PCR data** | | | **Zygosity** |
|  |  | **Positive** | **Negative** | **Total # plants** |  |
| 431-1-3-1 | BW^1^-A-11 | 18 | 0 | 18 | Homozygous^3^ |
| 431-5-1-1 | BW-B-4 | 15 | 0 | 15 | Homozygous |
| 446-1-1-1 | BW-F-10 | 22 | 0 | 22 | Homozygous |
| 426-2-1-4 | XC9^2^-104-1 | 19 | 0 | 19 | Homozygous |
| ^1^ Transgenic wheat generated in Bobwhite background  ^2^ Transgenic wheat generated in Xin Chun 9 background  ^3^ Homozygous lines were advanced to subsequent generations | | | | | |

| **Supplementary Table 3** Yield associated components collected from a population of homozygous transgenic lines | | | | | | |
| --- | --- | --- | --- | --- | --- | --- |
| Line name | Primary head size (cm) | | Seed count/head | | Seed weight/head (gm) | |
|  | Mean ± SEM | P-value | Mean ± SEM | P-value | Mean ± SEM | P-value |
| BW | 8.56 ± 0.08 | / | 36.2 ± 2.56 | / | 1.30 ± 0.08 | / |
| BW-A-11 | 9.24 ± 0.02 | 0.0147* | 38.6 ± 4.50 | 0.6553 | 1.46 ± 0.18 | 0.4390 |
| BW-B-4 | 9.14 ± 0.20 | 0.0296* | 27.6 ± 2.11 | 0.0320* | 1.18 ± 0.07 | 0.2936 |
| BW-F-10 | 8.40 ± 0.14 | 0.3718 | 36.6 ± 3.39 | 0.9272 | 1.34 ± 0.13 | 0.8011 |
| XC9 | 8.92 ± 0.15 | / | 39.6 ± 2.66 | / | 1.80 ± 0.13 | / |
| XC9-104-1 | 8.98 ± 0.17 | 0.8076 | 28.0 ± 2.86 | 0.0179* | 1.30 ± 0.12 | 0.0218* |
| * Indicates significant difference at P = 0.05 in the Student's *t*-test | | | | | | |
